# Supplementary material for: Structure of the ATP-driven methyl-coenzyme M reductase activation complex
Source: Nature. 2025 Apr 16;642(8068):814–21. doi: 10.1038/s41586-025-08890-7 (PMC12176620; doi:10.1038/s41586-025-08890-7)
Supplement: Supplementary file 1 — This file contains Supplementary Methods, Supplementary Fig. 1 and Tables 1–5. [file 41586_2025_8890_MOESM1_ESM.pdf]

nature portfolio

https://doi.org/10.1038/s41586-025-08890-7

Supplementary information

Structure of the ATP-driven methyl-coenzyme M reductase activation complex

In the format provided by the authors and unedited

Supplementary Information for

Structure of the ATP driven Methyl-coenzyme M reductase activation complex

Authors:

Fidel Ramírez-Amador[#1,2], Sophia Paul[#1,2], Anuj Kumar[#1,2], Christian Lorent[3], Sebastian Keller[4], Stefan Bohn[5], Thinh Nguyen[4], Stefano Lometto[6], Dennis Vlegels[6], Jörg Kahnt[6], Darja Deobald[7], Frank Abendroth[2], Olalla Vázquez[1,2], Georg Hochberg[2,6], Silvan Scheller[4], Sven T. Stripp[3,8], Jan Michael Schuller*[1,2]

Affiliations:

1 – Center for Synthetic Microbiology (SYNMIKRO), Marburg, Germany.

2 – Department of Chemistry, Philipps University of Marburg, Marburg, Germany.

3 – Technische Universität Berlin, Division of Physical Chemistry, Berlin, Germany.

4 – Department of Bioproducts and Biosystems, School of Chemical Engineering, Aalto University, Espoo, Finland.

5 – Helmholtz Munich Cryo-Electron Microscopy Platform, Helmholtz Munich, Neuherberg, Germany.

6 – Max Planck Institute for Terrestrial Microbiology, Marburg, Germany.

7 – Department Environmental Biotechnology, Helmholtz Centre for Environmental Research-UFZ, Leipzig, Germany.

8 – University of Potsdam, Institute of Chemistry, Potsdam, Germany.

# These authors contributed equally.

* Correspondence to: jan.schuller@synmikro.uni-marburg.de

Supplementary information – Table of contents

Supplementary Methods – Chemical synthesis of coenzyme B p. 3

Supplementary Table 1 – List of primers p. 4

Supplementary Table 2 – List of plasmids p. 4

Supplementary Table 3 – Protein components determined by MS p. 4

Supplementary Table 4 – Metal quantification p. 5

Supplementary Table 5 – Bond metrics comparison between FeS clusters p. 5

Supplementary Figure 1 – Uncropped gels p. 6

Supplementary Methods

Chemical synthesis of coenzyme B

First, O-Phospho-L-threonine (250 mg, 1.3 mmol, 1 eq.) was dissolved in 5 mL of a 50/50 (w/v) dimethylformamide/H_2O mixture under stirring, then treated dropwise with tri-n-butylamine (3.9 mmol, 956 µL, 3.3 eq.) at 4°C. After complete dissolution, the solution was evaporated under high vacuum at room temperature until a sticky consistency was obtained. 7,7'-Dithiodiheptanoic acid NHS-ester (974 mg, 1.9 mmol, 1.5 eq.) was added in one portion, followed by anhydrous dimethylformamide to achieve a clear solution (~5 mL). Triethylamine (408 mg, 4 mmol, 3 eq.) was then added and the solution was stirred for 5 h at room temperature. The resulting solution was divided equally into 50 mL conic plastic centrifuge tubes and diluted with pre-chilled (4°C) lithiumperchlorate-ethylacetate solution (6g/100 ml), before vortexing. After standing for 10 min at -20°C the tubes were centrifuged (4000 rpm) and the white precipitate was collected and washed 3 times with acetone (50 mL). Subsequently, the precipitate was dried and resuspended in 25 mL of a freshly prepared 500 mM TCEP solution, adjusted to pH 7 with aqueous sodium hydroxide, and stirred for 2 h at room temperature. This solution was loaded onto a pre-equilibrated flash column (Büchi Flashpure Ecoflex C18, 120g) with H_2O containing 0.1% formic acid, and the product was eluted using a linear gradient from H_2O to acetonitrile (both with 0.1% formic acid) over 50 min. Fractions containing the crude product were snap-frozen and lyophilized. The resulting white powder was further purified by preparative HPLC (Nucleodur C18 HTec, 5 µm, 250*21, Machery-Nagel) using a linear gradient from H_2O to acetonitrile (both with 0.05% trifluoracetic acid) over 50 min. Product-containing factions were immediately snap-frozen, aliquoted, and lyophilized. This procedure was repeated once more, yielding 222 mg in total (647 µmol, 50% yield), which was stored at -80 °C under nitrogen atmosphere.

Supplementary Table 1. List of primers.

Name	Description	Primer seq (5'-3')
FRM100_Fwd	To anneal the designed *mcrC*-gRNA and clone it into the plasmid pMM002p	AGATTACCTACTGGCATAAAATCACTTA
FRM101_Rev		TATCTAAGTGATTTTATGCCAGTAGGTA
FRM127_Fwd	To clone TS-tagged *mcrC* into the plasmid pMM002p by Gibson assembly (overhangs in bold) and plasmid sequencing	**TATAGTTATATGATAATTTAATAAAATTCG**GATATTTTAGAGTACGAAACTGGTTTACCT
FRM128_Rev		**TTATATTTTGATCGATCAGCTGAATTAACG**TGCGTAGTCTTCTACGAAGTCCATTTCTTC
FRM126_Fwd	For sequencing TS-tagged *mcrC* in the genome of *M. maripaludis*	GATGTCAGCGTGGTCGCA
SP026_Rev		GAGCAGGAGCAAAGTACATTGA

Supplementary Table 2. List of plasmids.

Name	Description	Reference
pMM002p	Plasmid carrying the CRISPR/*Lb*Cas12a machinery for genetic modification of *M. maripaludis*	Bao *et al.* [20]
pMM002p/gRNA	Plasmid carrying the CRISPR/*Lb*Cas12a and gRNA sequence targeting to *mcrC*	This study
pMM002p/TS-*mcrC*	Plasmid for the insertion of a TS-tagged version of *mcrC* into the genome of *M. maripaludis*	This study

Supplementary Table 3. Protein components present in the MCR activation complex determined by MS.

Name	Description	Mol. Size (kDa)	Uniprot ID
McrA	Methyl-coenzyme M reductase subunit alpha	61.1	A0A2L1CBB0
A2	Methyl-coenzyme M reductase system component A2	59.5	A0A2L1C9A1
Mmp3	Methanogenesis marker protein 3	56.4	A0A2L1CAI0
McrB	Methyl-coenzyme M reductase subunit beta	46.7	A0A2L1CBB3
Mmp7	Methanogenesis marker protein 7	34.9	A0A2L1C9H0
McrG	Methyl-coenzyme M reductase subunit gamma	29.6	A0A2L1CBG2
McrC	Methyl-coenzyme M reductase operon protein C	21.3 (24.3)[a]	A0A2L1CBQ8
Mmp17	Methanogenesis marker protein 17	21.1	A0A2L1C8U1
DUF2098	Domain of unknown function-containing protein 2098	10.6	A0A2L1CAX0

[a] overall molecular weight when fused to the Twin-Strep (TS) tag.

Supplementary Table 4. Metal quantification via ICP-QQQ-MS analysis from two independent batches of purified MCR activation complex from *M. maripaludis*. Values are calculated based on a predicted molecular weight of 478 kDa, according to the Extended Data Table 3 and the architecture $Mcr(ABG)_2$ + A2 + Mmp3 + Mmp7 + McrC + Mmp17 + DUF2098 observed in the protein structure. *Protein concentration in batch 1 = 10 µM; Protein concentration in batch 2 = 7 µM; NE = Not Expected; NT = Not Tested*

Element	Batch 1 (mol/mol protein)	Batch 2 (mol/mol protein)	Expected
^{56}Fe	25.4	28.7	24[a]
^{58}Ni	1.6	1.8	2[b]
^{61}V	0	NT	NE
^{66}Zn	4	10.9	1[c]
^{95}Mo	0.5	1.2	NE

[a] from three [8Fe-9S-C] clusters.
[b] from two F_{430} molecules.
[c] from A2 component. More Zn ions potentially bind, but these were neither observed in our cryoEM maps nor predicted from the protein sequences.

Supplementary Table 5. Bond metrics averages comparison between the FeS clusters detailed in this study and similar topologies.

Bond / Å	FeS[a]	FeFeco (8BOQ)	FeMoco (3U7Q)	Fe_8S_8 (3PDI)	K-cluster (7BI7)
Fe-C	1.99 ± 0.01	2.00 ± 0.01	1.99 ± 0.01	NA[b,e]	NA[b]
Fe-S	2.25 ± 0.03	2.26 ± 0.04	2.25 ± 0.03<br>(2.36 ± 0.01)[c]	2.28 ± 0.02	2.36 ± 0.1
Fe-Fe	2.61 ± 0.03	2.62 ± 0.03<br>(2.82 ± 0.09)[d]	2.64 ± 0.03<br>(2.69 ± 0.029)[d]	2.65 ± 0.01	2.69 ± 0.11

[a] this study
[b] not applicable.
[c] Mo-S.
[d] Fe8-Fe (see Extended Data Fig. 8b).
[e] published before demonstrating the presence of a central carbide ion.

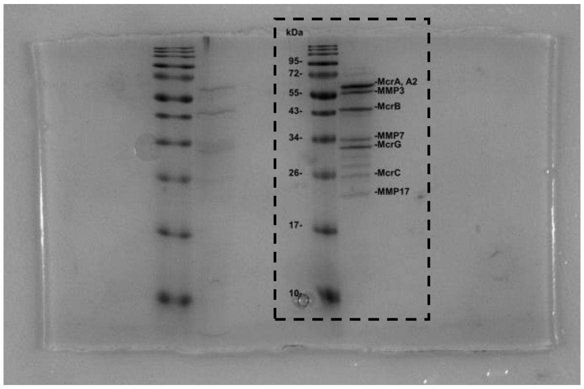

Fig. 1a (top)

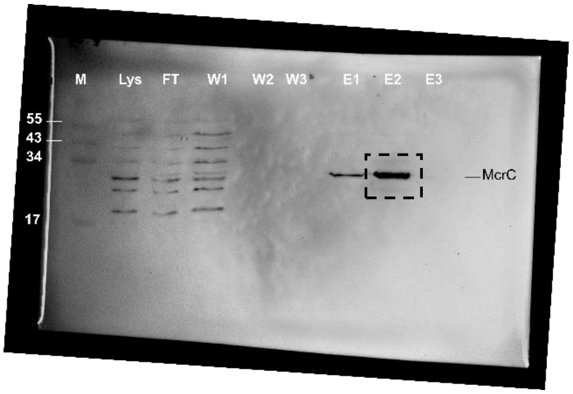

Fig. 1a (bottom)

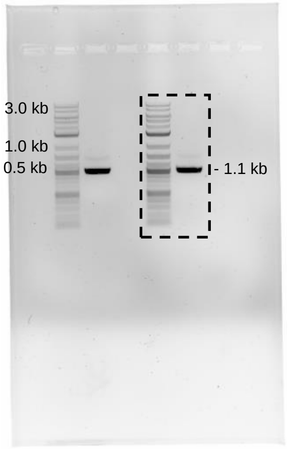

Ext. Data Fig. 1c

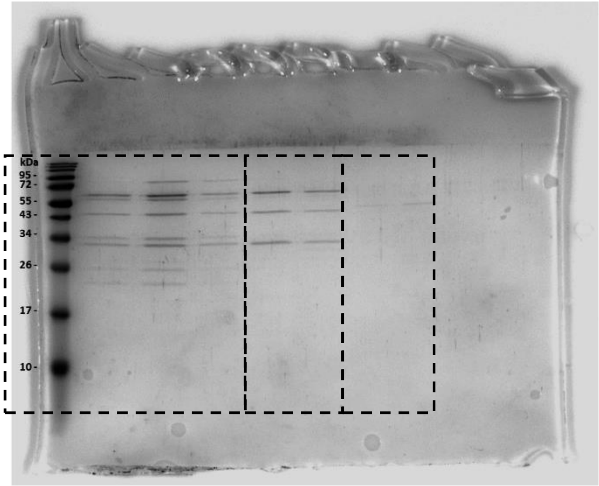

Ext. Data Fig. 2c

Supplementary Fig. 1 | Uncropped gels. Cropped regions shown in the main text are those indicated within dashed squares.
