# Supplementary material for: Structure of the ATP-driven methyl-coenzyme M reductase activation complex
Source: Nature. 2025 Apr 16;642(8068):814–21. doi: 10.1038/s41586-025-08890-7 (PMC12176620; doi:10.1038/s41586-025-08890-7)
Supplement: Supplementary file 2 — Reporting Summary [file 41586_2025_8890_MOESM2_ESM.pdf]

# nature portfolio

Corresponding author(s): Jan M. Schuller

Last updated by author(s): Feb 24, 2025

# Reporting Summary

Nature Portfolio wishes to improve the reproducibility of the work that we publish. This form provides structure for consistency and transparency in reporting. For further information on Nature Portfolio policies, see our Editorial Policies and the Editorial Policy Checklist.

## Statistics

For all statistical analyses, confirm that the following items are present in the figure legend, table legend, main text, or Methods section.

| n/a | Confirmed | |
|---|---|---|
| ☐ | ☒ | The exact sample size (*n*) for each experimental group/condition, given as a discrete number and unit of measurement |
| ☐ | ☒ | A statement on whether measurements were taken from distinct samples or whether the same sample was measured repeatedly |
| ☐ | ☒ | The statistical test(s) used AND whether they are one- or two-sided<br>*Only common tests should be described solely by name; describe more complex techniques in the Methods section.* |
| ☒ | ☐ | A description of all covariates tested |
| ☒ | ☐ | A description of any assumptions or corrections, such as tests of normality and adjustment for multiple comparisons |
| ☐ | ☒ | A full description of the statistical parameters including central tendency (e.g. means) or other basic estimates (e.g. regression coefficient) AND variation (e.g. standard deviation) or associated estimates of uncertainty (e.g. confidence intervals) |
| ☐ | ☒ | For null hypothesis testing, the test statistic (e.g. *F*, *t*, *r*) with confidence intervals, effect sizes, degrees of freedom and *P* value noted<br>*Give P values as exact values whenever suitable.* |
| ☒ | ☐ | For Bayesian analysis, information on the choice of priors and Markov chain Monte Carlo settings |
| ☒ | ☐ | For hierarchical and complex designs, identification of the appropriate level for tests and full reporting of outcomes |
| ☒ | ☐ | Estimates of effect sizes (e.g. Cohen's *d*, Pearson's *r*), indicating how they were calculated |

*Our web collection on statistics for biologists contains articles on many of the points above.*

## Software and code

Policy information about availability of computer code

| | |
|---|---|
| Data collection | CryoEM micrographs: Thermo Scientific EPU 3 software; Size exclusion chromatography data: Chromeleon v7.2.1; Mass photometry data: AcquireMP v2.3; Gas chromatography data: OpenLab CDS and TotalChrom v.6.3.4; Mass spectrometry data: Byonic (MS) and MassHunter 4.2 (ICP-MS). |
| Data analysis | CryoEM data: cryoSPARC v4; Protein structure and imaging: UCSF ChimeraX 1.6.1 and UCSF Chimera 1.17.3; Model building and refinement: Coot, PHENIX and eLBOW packages and tools; Prediction of structural models: AlphaFold 2and ModelAngelo v.1.0; Quality assessment of the protein model: MOLPROBITY; Size exclusion chromatography data: Chromeleon 7.2.1; Mass photometry data: DiscoverMP v2.3; Gas chromatography data: OpenLab CDS and TotalChrom v.6.3.4; Phylogenetic analysis: MAFFT, trimAI, UFBoot2, IQ-TREE2. Electronic spectrum data: Global Gauss Fit v.7. DNA sequencing: SnapGene 8.0. |

For manuscripts utilizing custom algorithms or software that are central to the research but not yet described in published literature, software must be made available to editors and reviewers. We strongly encourage code deposition in a community repository (e.g. GitHub). See the Nature Portfolio guidelines for submitting code & software for further information.

## Data

Policy information about availability of data

All manuscripts must include a data availability statement. This statement should provide the following information, where applicable:

- Accession codes, unique identifiers, or web links for publicly available datasets
- A description of any restrictions on data availability
- For clinical datasets or third party data, please ensure that the statement adheres to our policy

The data that support this study are available from the corresponding author upon request. The cryo-EM maps reported on this article are available in the Electron Microscopy Data Bank (EMDB) with the accession codes EMD-51767 (MCR activation complex + A2 after incubation with ATP) [https://www.ebi.ac.uk/emdb/EMD-51767], EMD-19787 (MCR activation complex as isolated + A2) [https://www.ebi.ac.uk/emdb/EMD-19787] and EMD-19788 (MCR activation complex as isolated without A2) [https://www.ebi.ac.uk/emdb/EMD-19788]. The local refined mask maps for A2, FeS containing subunits and Mmp3 were submitted along with EMD-51767 and EMD-19787. The atomic models are deposited in the Protein Data Bank (PDB) under the codes 9H1L (MCR activation complex + A2 after incubation with ATP) [https://doi.org/10.2210/pdb9h1l/pdb], 8S7V (MCR activation complex as isolated + A2) [https://doi.org/10.2210/pdb8s7v/pdb] and 8S7X (MCR activation complex as isolated without A2) [https://doi.org/10.2210/pdb8s7x/pdb]. Structural data used for comparison are available in the Protein Data Bank under the codes 3M32 (MCR from M. marburgensis bound to CoMS-SCoB) [https://doi.org/10.2210/pdb3m32/pdb], 1HBM (MCR from M. thermautotrophicus bound to CoMS-SCoB) [https://doi.org/10.2210/pdb1hbm/pdb], 8CRS (cryoEM structure of Mo-nitrogenase from A. vinelandii) [https://doi.org/10.2210/pdb8crs/pdb], 8DPN (cryoEM structure of Mo-nitrogenase from A. vinelandii DJ) [https://doi.org/10.2210/pdb8dpn/pdb], 6FEA (V-nitrogenase from A. vinelandii) [https://doi.org/10.2210/pdb6fea/pdb], 8OIE (cryoEM structure of Fe-nitrogenase from R. capsulatus) [https://doi.org/10.2210/pdb8oie/pdb], 8BOQ (Fe-nitrogenase from A. vinelandii) [https://doi.org/10.2210/pdb8boq/pdb], 3U7Q (Mo-nitrogenase from A. vinelandii at atomic resolution), [https://doi.org/10.2210/pdb3u7q/pdb], 3PDI (precursor-bound NifEN from A. vinelandii DJ) [https://doi.org/10.2210/pdb3pdi/pdb], and 7BI7 (P-cluster-bound NifB from M. thermoautotrophicus). Sequences for comparison with the A2 component are available in Uniprot with the accession numbers A0A8T3W5C7, D9PXN7, D7DTW8, Q7LYR5, Q58639, Q8TIZ1, A0A0E3R7T8, A0A0E3N9D1, F8ANN2, A6USB5, A0A8J8F9M9, and A0A8S9VY72. Activity assays measurements, size exclusion chromatography and spectroscopic data have been provided as Source Data. Original sequence alignments and phylogenetic trees have been deposited into Edmond, the Open Research Data Repository of the Max Planck Society, for public access and available under https://doi.org/10.17617/3.UZSFCW. All data needed to evaluate the conclusions in the paper are present in the paper and/or the supplementary information.

## Research involving human participants, their data, or biological material

Policy information about studies with human participants or human data. See also policy information about sex, gender (identity/presentation), and sexual orientation and race, ethnicity and racism.

| | |
|---|---|
| Reporting on sex and gender | N/A |
| Reporting on race, ethnicity, or other socially relevant groupings | N/A |
| Population characteristics | N/A |
| Recruitment | N/A |
| Ethics oversight | N/A |

Note that full information on the approval of the study protocol must also be provided in the manuscript.

# Field-specific reporting

Please select the one below that is the best fit for your research. If you are not sure, read the appropriate sections before making your selection.

☒ Life sciences ☐ Behavioural & social sciences ☐ Ecological, evolutionary & environmental sciences

For a reference copy of the document with all sections, see nature.com/documents/nr-reporting-summary-flat.pdf

# Life sciences study design

All studies must disclose on these points even when the disclosure is negative.

| | |
|---|---|
| Sample size | Sample size was not predetermined by statistical methods but three biological sample sizes and three technical replicates were used for in vitro analysis to provide datasets that enable statistics for the relevant experiments. The in vitro assays are tried-and-tested procedures that have been successfully performed by the authors in their laboratories on a day-to-day basis. The sample sizes were diligently chosen and accurately illustrate the differences throughout the various conditions. For cryoEM, two datasets containing 30,000 micrographs (incubated with ATP and 25º tilted) and 17,548 micrographs (sample as isolated, 20° tilted and non-tilted combined) were used in order to have a sufficient number of MCR's activation complex particles - with and without the A2 component - resulting in 3D reconstructions with a resolution of 2.1 (+A2 component, incubated with ATP), 2.56 (+A2 component, as isolated) and 2.78 Angstrom (-A2 component, as isolated). |
| Data exclusions | For strictly anaerobic enzymes, oxygen contamination cannot always be excluded and leads to a significant effect the experiments. If a measurement in the activity assays was obviously differing from the other replicates, this measurement was excluded from the dataset. For |

cryoEM, particles visibly containing the activation complex and/or the A2 component were chosen for determination of the high resolution structures.

| | |
|---|---|
| Replication | Enzymatic assays were performed from three independent biological replicates with at least three technical repetitions each. Spectroscopic data was obtained from three independent biological replicates. Sample separation via polyacrylamide gels and size exclusion chromatography was performed at least three times from independent biological repetitions, whereas mass photometry took place from two independent biological repetitions. All replication attempts were successful. For cryoEM, one dataset of 30,000 micrographs (incubation with ATP) and a second one of 17,548 micrographs (as isolated) were analyzed. |
| Randomization | Resolution determination was carried out with two independent half sets randomly selected by the cryoSPARC processing software according to the Gold-Standard FSC procedure. For all other experiments, randomization was not applicable, since in microbiological assays all parameters are tightly controlled and therefore covariates are not relevant to our study. |
| Blinding | Blinding was not relevant to our study since no higher order species were used. Moreover we have repeated experiments from three independent biological replicates with at least three technical replicates. |

# Reporting for specific materials, systems and methods

We require information from authors about some types of materials, experimental systems and methods used in many studies. Here, indicate whether each material, system or method listed is relevant to your study. If you are not sure if a list item applies to your research, read the appropriate section before selecting a response.

## Materials & experimental systems

| n/a | Involved in the study | |
|---|---|---|
| ☐ | ☒ | Antibodies |
| ☒ | ☐ | Eukaryotic cell lines |
| ☒ | ☐ | Palaeontology and archaeology |
| ☒ | ☐ | Animals and other organisms |
| ☒ | ☐ | Clinical data |
| ☒ | ☐ | Dual use research of concern |
| ☒ | ☐ | Plants |

## Methods

| n/a | Involved in the study | |
|---|---|---|
| ☒ | ☐ | ChIP-seq |
| ☒ | ☐ | Flow cytometry |
| ☒ | ☐ | MRI-based neuroimaging |

## Antibodies

| | |
|---|---|
| Antibodies used | StrepMAB-Classic HRP (1.5 mg/mL) IBA Lifesciences, Germany/2-1509-001<br>Conjugated monoclonal antibody to detect Strep-tag®II and Twin-Strep-tag® fusion proteins |
| Validation | StrepMAB-Classic HRP (1.5 mg/mL, IBA Lifesciences, Germany/2-1509-00 counts with a Bioz Stars standard score of 93/100, supported by multiple citations in journals with an impact factor equal or higher than 10 that have reported its use in western-blot experiments. This is according to the information from https://www.iba-lifesciences.com/strepmab-classic-hrp/2-1509-001 |

## Plants

| | |
|---|---|
| Seed stocks | *Report on the source of all seed stocks or other plant material used. If applicable, state the seed stock centre and catalogue number. If plant specimens were collected from the field, describe the collection location, date and sampling procedures.* |
| Novel plant genotypes | *Describe the methods by which all novel plant genotypes were produced. This includes those generated by transgenic approaches, gene editing, chemical/radiation-based mutagenesis and hybridization. For transgenic lines, describe the transformation method, the number of independent lines analyzed and the generation upon which experiments were performed. For gene-edited lines, describe the editor used, the endogenous sequence targeted for editing, the targeting guide RNA sequence (if applicable) and how the editor was applied.* |
| Authentication | *Describe any authentication procedures for each seed stock used or novel genotype generated. Describe any experiments used to assess the effect of a mutation and, where applicable, how potential secondary effects (e.g. second site T-DNA insertions, mosiacism, off-target gene editing) were examined.* |
